# Screening of Host Specific Lactic Acid Bacteria Active Against *Escherichia coli* From Massive Sample Pools With a Combination of *in vitro* and *ex vivo* Methods

**DOI:** 10.3389/fmicb.2019.02705

**Published:** 2019-11-21

**Authors:** Hao Ren, Eva-Maria Saliu, Jürgen Zentek, Farshad Goodarzi Boroojeni, Wilfried Vahjen

**Affiliations:** Institute of Animal Nutrition, Freie Universität Berlin, Berlin, Germany

**Keywords:** probiotics, lactic acid bacteria, host-derived, effective screening, *E. coli*, *ex vivo* model, massive sample pool

## Abstract

A novel three-step combination of *in vitro* and *ex vivo* screening was established to massively screen host derived lactic acid bacteria (LAB) from the broiler chicken intestine with inhibitory activity against *Escherichia coli*. In a first step, a massive sample pool consisting of 7102 broiler-derived colonies from intestinal contents were established and sub-cultured. Supernatants thereof were incubated with an *E. coli* model strain to screen suitable isolates with inhibitory activity. A total of 76 isolates of interest were subsequently further studied based on either pH dependent or -independent activity in the second step of the assay. Here, in-depth growth inhibition of the *E. coli* model strain and the potential of isolates for lactic acid production as inhibitory substance were indexed for all isolates. Resulting scatter plots of both parameters revealed five isolates with exceptional inhibitory activity that were further studied under *ex vivo* condition in the third step of the assay. These isolates were taxonomically classified as strains of the species *Lactobacillus agilis*, *Lactobacillus salivarius*, and *Pediococcus acidilactici*. Samples from the broiler chicken intestine were inoculated with the *Lactobacillus* isolates and the *E. coli* model strain. After 8 and 24 h incubation, respectively, growth of the *E. coli* model strain was monitored by cultivation of the *E. coli* strain in antibiotic supplemented medium. By their superior inhibitory activity against the *E. coli* model strain, one *L. agilis* and one *L. salivarius* strain were selected and characterized for further application as probiotics in broiler chicken. Additionally, their antibiotic resistance patterns and resilience under gastric stress of isolates were also characterized. The results of this study demonstrate that the novel isolation procedure was able to efficiently and rapidly isolate and identify bacterial strains from a massive sample pool with inhibitory potential against specific types of bacteria (here *E. coli*). The introduction of the final *ex vivo* selection step additionally confirmed the inhibitory activity of the strains under conditions simulating the intestinal tract of the host. Furthermore, this method revealed a general potential for the isolation of antagonistic strains that active against other pathogenic bacteria with specific biomarker.

## Introduction

The search for alternatives to antibiotics is an important topic worldwide. Various groups of feed additives have been studied so far and probiotics seem to be promising candidates to increase animal health and performance in the absence of in-feed growth promoters (Mehdi et al., 2018).

As defined by Food and Agriculture Organization/World Health Organization (FAO/WHO), probiotics are “Live microorganisms which when administered in adequate amounts confer a health benefit on the host” (Fao-Who., 2006). However, in the field of animal nutrition, especially for farm animals, probiotics are to protect the animal against specific pathogenic bacteria or have beneficial effects on animal performance (Chaucheyras-Durand and Durand, 2010; Liao and Nyachoti, 2017; Markowiak and Śliżewska, 2018).

As a group of extensively studied probiotic, lactic acid bacteria (LAB) have demonstrated inhibitory effects on certain microorganisms and potentially benefits on animal health (Dowarah et al., 2017). A large body of evidence have shown that LAB strains can exert beneficial impact by regulating intestinal inflammation or decreasing colonization of zoonotic bacteria like *Escherichia coli*, *Campylobacter jejuni* or *Salmonella enterica* (Santini et al., 2010; Vasanth et al., 2015; Azizkhani and Tooryan, 2016; Forkus et al., 2017; Wang et al., 2017). Among investigated pathogens, *E. coli* is one of the most well-documented target, and numerous investigations show efficiency of LAB on inhibiting *E. coli* growth or preventing *E. coli* infection (Sherman et al., 2005; Kimble et al., 2015; Azizkhani and Tooryan, 2016). Therefore, LAB have been also intensively studied and widely used in recent decades for their beneficial properties as potential antagonists (Kajander et al., 2005; Hong et al., 2014; Lan et al., 2016). Diverse LAB products have been developed on the basis of wide array of species including *L. reuteri*, *L. acidophilus*, *L. intestinalis*, *L. plantarum*, *L. casei*, and *L. sakei* (Kılıç and Karahan, 2010; Karami et al., 2017; Tashakor et al., 2017). The actual isolation of probiotic bacteria is a field of research that has not been addressed in depth so far. Theoretical selection criteria for probiotics including LAB for human use recommended by the WHO include host-related stress resistance, epithelial adhesion and antibacterial activity as well as biosafety (Zhang et al., 2016; de Melo Pereira et al., 2018). Other parameters such as aggregative ability, hydrophobic phenotyping, reduction of pathogenic virulence, immunomodulation and specific metabolic pathway were also reported as possible criteria for selection (Saint-Cyr et al., 2016). *In vitro* criteria are preferred because of simplicity and cost-efficiency (Papadimitriou et al., 2015). However, the characterization of probiotic LAB strains by using *in vitro* methods alone may not be sufficient to predict their *in vivo* scenario, as different bacterial strains may behave differently under the conditions of the intestinal tract (Murima et al., 2014). Whether the selected LAB are able to colonize the host is as well an essential question. On the other hand, *in vivo* selection procedures are time-consuming, costly and carry ethical considerations, even though it offers the most direct impact of probiotic on host animals at given condition (Martins et al., 2008). This implies that an efficient screening assay for potential probiotic bacteria should include the steps to mimic *in vivo* conditions and at the same time be feasible in terms of laboratory work.

Commonly, the number of isolates screened for probiotic activity were comparably low, ranging between 14 and 1150 isolates with the majority of studies using only 50 to 80 isolates (Robyn et al., 2012; Babot et al., 2014). Thus, to our best knowledge, there is no published method to massively screen bacterial isolates with specific antibacterial activity. Considering the vast diversity of bacterial species in the intestinal tract as well as the occurrence of numerous strains in each species, it seems promising to screen as many potential probiotic isolates as possible to increase the probability of success. Also, the origin of probiotic has not yet been considered as significant factor previously. However, the advantage of isolation of host-specific probiotics become increasingly focused because those strains have already shown the capability to colonize the hosts (Zmora et al., 2018).

Most existing studies on probiotic LAB focus solely on their antagonistic activity in *in vitro* (Gram and Ringø, 2005). Recently, it was hypothesized that the intestinal tract of poultry harbors strains capable to inhibit the inhabitation of potential pathogens (Nhung et al., 2017; Shang et al., 2018). Therefore, the present study developed a three-step combination of *in vitro* and *ex vivo* methods to massively screen LAB isolates for their potential to inhibit *E. coli*. The final *ex vivo* model confirmed inhibiting activity under conditions simulating the gastro intestinal tract simultaneously as it is more easily controlled. Due to the technical simplicity of this method, it has the general potential for the development of other probiotics that target specific bacteria.

## Materials and Methods

### Strains and Media

Throughout the study, an extended-spectrum beta-lactamase producing *E. coli* strain ESBL10716 (phylotype B1) was used as a model strain. It was isolated from excreta samples of broiler chicken by the Institute of Microbiology and Epizootics of Freie Universität Berlin within the RESET program and produces the CTX-M-15 lactamase (Falgenhauer et al., 2016). The resistance of model strain against cefotaxime was used as a specific marker in all culture and growth experiments. The strain was selected as a representative target strain from 13 *E. coli* strains of broiler origin in a pre-experiment, showing the strongest resistance against *in vitro* GIT stress and stress of random LAB supernatants (data not shown). The strain was stored as cryo stock and cultured in brain heart infusion broth (BHI, Carl Roth GmbH + Co., KG, Germany) for further application.

### Sampling and Original Isolation

Intestinal samples were taken from broiler chicken (Cobb500). Fresh digesta samples from the crop, ileum, jejunum and cecum and excreta were obtained from different feeding trials conducted at the Institute of Animal Nutrition, Freie Universität Berlin and immediately processed. The animals received standard basal feed with no zootechnical feed additives. Samples were serially diluted in Phosphate Buffered Saline (PBS, Sigma-Aldrich, Chemie GmbH, Germany) buffer, pH 7.4 and plated on de Man, Rogosa, and Sharpe (MRS, Carl Roth GmbH + Co., KG, Germany) agar plates. After anaerobic growth at 39°C for 48 h, single colonies from different dilutions and with different colony morphologies were picked with sterile toothpicks into microtiter plates supplemented with MRS broth (Carl Roth GmbH + Co., KG, Germany). Supernatants of colonies with visible growth were subcultured in microtiter plates. The original plates were kept at 4°C until after the preliminary screening (max. 48 h). Isolates of interest after the first screening were preserved from microtiter plates to cryo stock in −80°C freezer.

### Ethical Statement

Samples were taken from studies that were conducted in accordance with the German Animal Welfare Act (TierSchG) and approved by the local state office of occupational health and technical safety “Landesamt für Gesundheit und Soziales, Berlin” (LaGeSo Reg. Nr. T 0162/16 and A 0100/13).

### Step 1: Massive Isolation and Preliminary Screening of Intestinal Lactobacilli

In the first step, a large samplepool was established and subjected to a pre-screening system.

### Buffering and Deacidification Filtering (Pre-screening)

Regarding the initial pre-screening, two different approaches were tested to rapidly screen a large number of isolate supernatants. Thus, before inoculation of the *E. coli* model strain, one subset of supernatants (2208 isolates) was mixed with same volume of double strength BHI medium buffered with 0.4 M citrate buffer (pH = 6.2, Sigma-Aldrich, Chemie GmbH, Germany), while another subset of supernatants (2592 isolates) was supplemented with 3.5 μL 5 M NaOH (Carl Roth GmbH + Co., KG, Germany). Optimal buffering and deacidification conditions that still allowed growth of the *E. coli* model strain were determined in a series of pre-experiments (results shown in Supplementary Materials). The microtiter plates were inoculated with 10 μL *E. coli* culture (10^4^ CFU/mL and incubated overnight aerobically at 37°C. Final optical density (OD) was read with a microtiter plate reader at 690 nm (Tecan Infinite200Pro, Germany) to determine bacterial growth. The final OD was used as indicator of inhibitory potential of a given isolate.

### Step 2: *In vitro* Selection

The second step of the screening studied the *E. coli* growth inhibition in depth via growth curves in combination with lactic acid production of the isolates as probable inhibitory substance.

For this purpose, supernatants were generated by inoculating the LAB candidates at 10^4^ CFU/mL in 10 mL MRS medium and incubated anaerobically at 39°C for 48 h. Supernatants were either used as is or adjusted to pH 6.5 with 5 M NaOH.

Lag time for *E. coli* growth was chosen as the first inhibition-related parameter and assessed according to previous study with necessary modification. In brief, pH-neutralized supernatants of the isolates were combined with same volume of double strength BHI medium and then dispensed into microtiter plates at 190 μL per well. The model *E. coli* strain (10 μL) was added to each well yielding a final concentration of 10^4^ *E. coli* cells/mL. Cultures were then incubated aerobically at 37°C and turbidity (OD_690__nm_) was recorded every 5 min for 24 h using a microtiter plate reader (Tecan Infinite200Pro, Germany). Resulting growth curves were analyzed for lag time against respective controls without supernatants using the 3-parameter sigmoidal equation for bacterial growth and compared to respective controls. All growth experiments were carried out in triplicate. Lactic acid production was measured as aother probable inhibitory parameter. Triplicates of non-pH controlled supernatants were prepared as described above. Protein was precipitated by Carrez solution, the supernatant was filtered (0.45 μm filter, Carl Roth GmbH + Co., KG, Germany) and the concentration of lactic acid was measured with an enzymatic test reagents (R-Biopharm AG, Germany) according to the manual with minor modification. The L-/D-lactic acid standards were prepared with diluting pure L-/D-lactic acid to a serial dilutions (0, 26.5, 53, 79.5, 132.5, 185.5, 238.5, and 265 mg/L) and treated supernatant of each isolate was 1:50 diluted. 10 μL of each sample was added to 200 μL reagent 1 (L-/D-lactic acid-dehydrogenase buffer) and incubated at room temperature for 3 min. 10 μL distilled water was also incubated as reagent blank (RB). The OD was read once as A_1_ after the incubation, then 50 μL reagent 2 (NAD solution) was added to each reaction. The samples were again incubated in room temperature for 15 min, then the absorbance was measured again as A_2_. The standard curve was established with adjusted OD absorbance of all standards with equation “ΔA = (A_2_-0.808A_1_)_Sample_-(A_2_-0.808A_1_)_RB__._” The standard curve for both L-lactic acid and D-lactic acid were plotted accordingly (calibration curves are shown in the Supplementary Figures 4, 5). The concentration of each sample was further calculated with their corresponding adjusted OD by the standard curve.

To make the data comparable, the results of lag time and concentrations of lactic acids were indexed as follows: each read of lag time and lactic acid concentration was divided by the maximum value of the data set (lag time _n_/lag time _max_ or lactic acid _n_/lactic acid _max_) to reflect individual lag time extension/lactic acid production level among all tested isolates. Supernatants with superior lag time- and lactic acid index were then introduced to the final step of the isolation assay.

### Step 3: *Ex vivo* Selection

An *ex vivo* model was prepared on the basis of a published method with minor modification (Starke et al., 2013) to test the impact of the chosen isolates on the survival of the *E. coli* model strain under conditions that are similar to the intestinal tract. Briefly, fresh digesta samples from the crop, jejunum or ileum were diluted 1:2 (w/v) with sterilized water. After sedimentation for 5 min, the supernatant of this suspension was transferred to sterile 15 mL tubes and dispensed into microtiter plates. LAB candidates (final concentration 10^7^ CFU/mL) and the *E. coli* model strain (final concentration 10^4^ CFU/mL) were then inoculated in triplicate. Non-inoculated suspensions served as controls. All suspensions were incubated anaerobically at 37°C. This lower temperature than under *in vivo* conditions was chosen to chosen to allow the *E. coli* strain a better survival and therefore better detectability, as results show that even at 37°C the most active isolates completely inhibited *E. coli* survival after 24 h. Samples (10 μL) were obtained after 8 and 24 h incubation, respectively, and inoculated into cefotaxime (8 μg/mL, Thermo Fisher GmbH, Germany) containing BHI agar plates. After growth, colony forming units (CFU) as well as growth curves were analyzed as described above.

**FIGURE 1:**
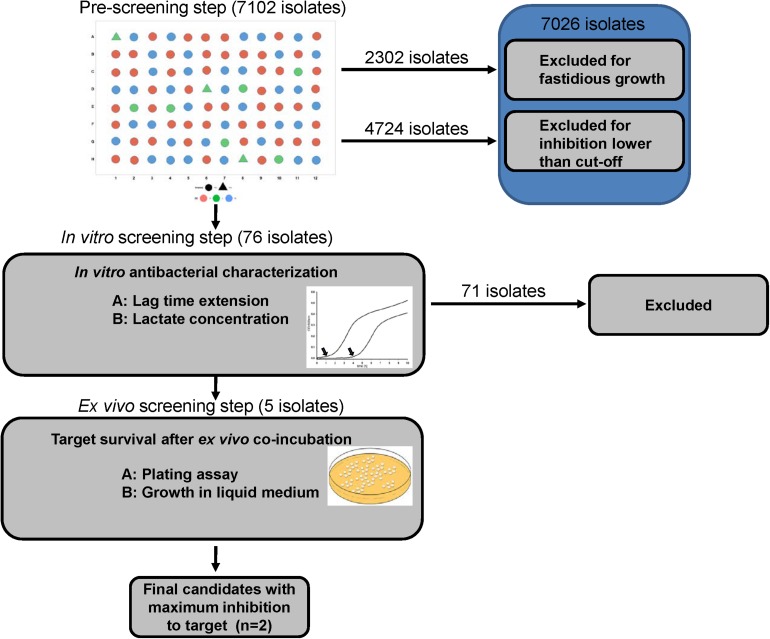
Experimental design of the assay.

The three consecutive steps of screening are schematically shown in Figure 1.

### Eligibility Criteria in Each Step

Isolates that showed inhibitory activity against the *E. coli* strain were determined after each step of the procedure. In the first step (massive screening), the end-point OD of *E. coli* culture co-incubated with pre-treated supernatants (buffered/deacidified) of LAB isolates were referred as the indicator to estimate growth inhibition of the *E. coli* strain. A cut-off value of 0.2 at OD_690__nm_, corrected for controls, was set as the threshold for growth inhibition. A total of 76 isolates were eligible for the next step.

In the second step, both lag time and lactic acid production were indexed. A scatter plot of these indices revealed those isolates with superior inhibition/concentration. The best five isolates were selected for the final step.

In the third step, isolates that induced the lowest *E. coli* survival in both growth assay and CFU after incubation under *ex vivo* conditions were selected as the final candidates.

### Characterization of Selected Candidates

#### Taxonomic Identification of Candidates

Selected LAB isolates showing successful inhibition were identified on the species level via full length 16S rDNA sequence analysis using the classic universal primer pair F27 and R1492 by DSMZ(German Collection of Microorganisms and Cell Cultures, Germany) according previously published method (Stackebrandt et al., 2002).

#### Production of SCFA

Short-chain fatty acid in the supernatants of the candidates was analyzed via gas chromatography (Agilent Technologies 6890 N coupled with auto sampler G2614A and auto injector G2613A; Santa Clara, CA, United States). A total of 500 μl of each sample was mixed with the same volume of a CuSO4 solution (0.5 mmol/L). Protein in samples were precipitated by addition of 200 μl Carrez solution and centrifugation. After centrifugation, the samples were filtered through a 0.45 μm cellulose acetate (CA) filter and diluted with 0.5 mmol/L CuSO4 by 1:40 (v/v) for measuring. SCFA were then determined with a previously published method with minor modification (Schäfer, 1995). An Agilent 19095N-123 HP-INNOWAX polyethylene glycol column was employed in this experiment.

#### Aggregation Assessment

The auto-/co-aggregation abilities of selected LAB isolates were evaluated by a reported method with minor modification (Collado et al., 2008). Briefly, for auto-aggregation, stationary phase cultures were centrifuged (3 min, 10000 *g*, 4°C) and washed three times in PBS. The centrifugates were then re-suspended in PBS to an OD_690__nm_ of 0.25 ± 0.05 (comparable to 10^7^ – 10^8^ cells/mL). Turbidity was measured every 2 h. To determine the auto-aggregation of strains, turbidity was calculated by the following equation: Auto-aggregation (%) = 1-(OD_t_-OD_0_) × 100 (where OD_t_ was absorbance after 2 h; OD_0_ was the initial absorbance). For the co-aggregation, the centrifugates of lactobacilli isolates and tested *E. coli* were both processed and prepared as described above. Lactobacilli isolates and model *E. coli* were mixed at equal volume (vol/vol). Turbidity was monitored as described above and the co-aggregation rate was calculated by: [(OD_E_ + OD_L_)/2-(OD_CO_)/(OD_ESBL_ + OD_LAB_)/2] × 100 (where OD_E_ was the *E. coli* control; OD_L_ was the lactobacilli isolate control and OD_CO_ expresses the turbidity of coincubation).

#### Tolerance of Isolates to Acid, Osmotic Pressure and Bile

The tolerance of lactobacilli isolates to acid and bile stress was assessed by their viability and growth under conditions encountered in the stomach and small intestine, respectively.

Overnight cultures were centrifuged (3 min, 10000 *g*, 4°C) and washed three times with PBS, pH 7.0. The centrifugates were diluted to 10^8^ cells/mL then inoculated into acidified MRS broth at pH 2, 3, and 4 or MRS broth supplemented with bile salt (w/v: 0.1%/0.3%/0.5%/0.7%) in a microtiter plate, respectively, and incubated anaerobically overnight at 39°C. Turbidity (OD_690__nm_) was monitored every 5 min and growth curves were plotted accordingly. Another set of centrifuges of the same cultures was diluted with pH-adjusted incubation buffer and bile containing buffer to approximately log_10_ 8.0 cells/mL and incubated at 39°C for 6 h. Samples from incubations were taken every 2 h and viable cells were enumerated by plating.

Tolerance against osmotic pressure was assessed with a published protocol with minor modification (Ng et al., 2015). After overnight incubation (anaerobically, 39°C), cultures were centrifugates (3 min, 10000 *g*, 4°C), washed in PBS buffer and 10^9^ cells/mL were inoculated in MRS broth supplemented with sodium chloride of 2–10% final concentration. End-point turbidity at OD_690__nm_ was determined after 40 h and compared to respective controls.

#### Adhesion and Competitive Adhesion Assay

The *in vitro* adhesion assay was performed according to previous report with minor modifications (Yeo et al., 2016). Caco-2 cells were cultured in Dulbecco’s modified Eagle’s medium/Ham’s Nutrient Mixture F-12 (DMEM/F-12, Merck, Germany) supplied with 10% fetal bovine serum (FBS), streptomycin (100 μg/mL), and amphotericin B (0.5 μg/mL) under 5% CO_2_ in a 95% air atmosphere with 90% humidity at 37°C. The cells were then seeded onto 12 well plates (Greiner Bio-one GmbH, Germany) with of 2 × 10^5^ cell per well in antibiotic free medium. After confluence of cells reached approximately 80%, the cells were exposed to 10^8^ CFU lactobacilli candidate or combination of 10^8^ CFU lactobacilli candidates with 10^7^ CFU model *E. coli*. After incubation at 37°C for 1.5 h, non-adhering bacteria were washed three times with PBS. The monolayer of cells was detached with cell scratcher and re-suspended with 500 μl PBS. After a serial dilution, detached cells were then plated onto MRS agar plates or BHI agar plates supplemented with 8 μg/ml cefotaxime. Adhesion and competitive adhesion of lactobacilli was determined by enumeration of colonies on agar plates and calculated as relative to controls.

#### Antibiotic Susceptibility

The minimum inhibitory concentration (MIC) of a selected panel of antibiotics including ampicillin, chloramphenicol, clindamycin, erythromycin, gentamycin, kanamycin, streptomycin, and tetracycline toward candidates were determined using a broth microdilution test as described by the Clinical and Laboratory Standards Institute (CLSI) with minor modification (CLSI, 2012). Selected candidates were incubated as described. Microdilution plates containing 100 μl MRS medium were inoculated with 50 μl inoculum as well as 50 μl antibiotic solution at appropriate concentration (0.25–128 μg/ml). Negative and positive controls were non-inoculated/inoculated wells without antibiotics. After anaerobic incubation at 37°C for 48 h, the MICs were determined as their lowest concentration capable to inhibit the visible bacterial growth. The reference strain DSM 20016 (*L. reuteri*) was used as the quality control. The cut-off value documented by European Food Safety Authority (EFSA, 2012) was used to categorize susceptibility or resistance of selected candidates.

### Statistical Analysis

The experiments were performed twice in triplicates for the determination and comparison in screening and characterization section. Results are presented as means ± standard deviation (SD). For *in vitro* data, lag times were modeled and analyzed by 3-parameter sigmoidal equation using SigmaPlot version 11 (Systat Software Inc., United States). Statistical significance of comparison in screening steps was assessed using Mann–Whitney test. Significance of different cell adhesion level was evaluated with Duncan’s multiple range test. Statistical procedures were performed at a significance level of 95%. All calculations were performed using the statistics software IBM SPSS (Version 22, Chicago, IL, United States).

## Results

### Step 1: Massive Isolation and Preliminary Screening of Intestinal Lactobacilli

In the initial screening step, 7102 colonies were processed. 2302 isolates failed to show growth after sub-culturing colonies in liquid medium. The remaining 4800 isolates were further tested for inhibitory activity against the model *E. coli* strain with the described buffering or deacidification treatments. Of those isolates, a total of 76 isolates showed either strong growth inhibition (OD_690__nm_ < 0.2) in buffered supernatants (48 of 2160 isolates tested, 2.2% positive) or in deacidified supernatants (28 of 2564 isolates tested, 1.1% positive).

### Step 2: *In vitro* Selection

**FIGURE 2:**
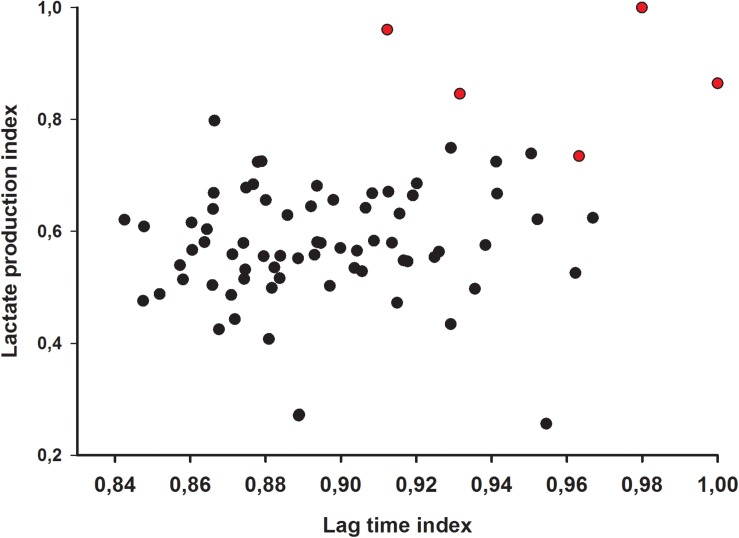
Lag time and lactic acid index of 76 lactic acid bacteria isolates. Red dots indicate selected candidate isolates.

A more in-depth evaluation of the inhibitory activity of isolates was studied by monitoring *E. coli* lag time lag time after incubation in supernatants. The production of lactic acid by the isolates was used as an additional inhibitory parameter, as lactic acid is strongly inhibitory to most enterobacteria. Increase of lag time of the *E. coli* strain in supernatants ranged from 1.17 h to 2.57 h and lactic acid production in overnight cultures ranged from 14.07 g/L to 16.01 g/L (Table 1). From the comprehensive comparison of both lag time and lactic acid production indices, five isolates were chosen for the final step (Figure 2).

**TABLE 1:** Lactic acid production of five lactic acid bacteria candidates and lag time of the *E. coli* model strain in media supplemented with supernatants of the candidates.

**Strain**	**Lactic acid (g/L)**	**Lag time (h)**
S1	15.06 ± 1.96	8.57 ± 1.16^∗^
S26	14.07 ± 4.35	8.01 ± 0.79
S62	15.30 ± 2.65	8.09 ± 0.79
S70	16.01 ± 3.08	8.69 ± 0.83^∗^
S73	15.46 ± 3.01	8.86 ± 1.39^∗^
Control	–	6.29 ± 0.87

*^∗^ = Significantly different to control (*p* ≤ 0.05, Mann–Whitney test).*

Detailed lag times of all 76 isolates were shown in Supplementary Table 1. Regarding the original selection, two of the five strains were isolated via the buffer system, while three strains were obtained from the deacidification treatment.

### Step 3: *Ex vivo* Selection

**FIGURE 3:**
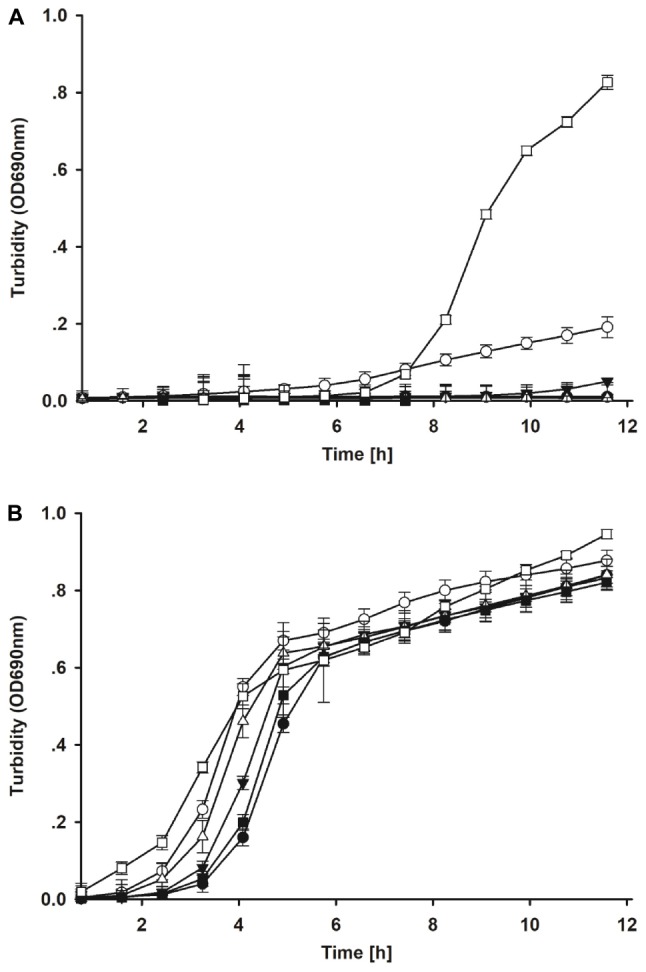
**(A)** Growth of the model *E. coli* strain after *ex vivo* co-incubation with candidate lactic acid bacteria isolates for 8 h in crop contents. Filled circle = S1; open circle = S26; filled down triangle = S62; open downward triangle open diamond = S70; filled square = S73; open square = control. **(B)** Growth of the model *E. coli* strain after *ex vivo* co-incubation with candidate lactic acid bacteria isolates for 8 h in jejunum contents. Filled circle = S1; open circle = S26; filled down triangle = S62; open downward triangle open diamond = S70; filled square = S73; open square = control.

In the *ex vivo* selection step, five isolates from the *in vitro* selection were co-incubated with the *E. coli* model strain in intestinal contents of broiler chicken. After 8 h co-incubation, most candidates showed a stronger inhibitory activity against the *E. coli* model strain in crop contents than in jejunum contents (Figures 3A,B). Strain S26 only led to reduced growth of the *E. coli* strain but all other strains resulted in complete inhibition in crop content. No inhibition by all strains was observed after 8 h in jejunum contents. When the *ex vivo* co-incubation was extended to 24 h and studied via CFU, the inhibitory effects of the candidate isolates were amplified (Table 2). These results indicate that candidate S1 and S73 completely reduced the survival of *E. coli* in intestinal contents.

**TABLE 2:** Survival of the *E. coli* model strain after 24 h incubation with lactic acid bacteria candidates in intestinal contents (CFU/g content).

	**Crop**	**Jejunum**
S1	ND	ND
S26	7.40 ± 0.67 × 10^3^	4.00 ± 0.54 × 10^4^
S62	3.10 ± 0.50 × 10^3^	6.80 ± 0.42 × 10^3^
S70	7.20 ± 0.80 × 10^2^	3.80 ± 0.54 × 10^2^
S73	ND	ND
*E. coli* control	3.80 ± 0.22 × 10^4^	9.10 ± 1.79 × 10^4^
Initial *E. coli* count	8.70 ± 1.35 × 10^4^	8.70 ± 1.35 × 10^4^
Negative control	ND	ND

*ND = not detected (detection limit: 10^2^ CFU/g content).*

### Characterization of Final Lactic Acid Bacteria Candidates

Data on the characterization of the Lactobacillus isolates is shown in Table 3. The final 2 candidates, *Lactobacillus* strains S1 (*L. salivarius*) and S73 (*L. agilis*) originated from ileum and crop samples of 42-day old broilers, respectively. Strain S1 was found using the buffer system, while S73 originated from the deacidification treatment. Strain S73 exhibited a stronger production of total SCFA in MRS medium than strain S1 (the production of lactic acid was included in Table 1). Regarding auto-aggregation, S73 showed a higher rate than S1. As to co-aggregation, no significant difference was observed in co-aggregative ability with the indicator *E. coli* strain after 24 h incubation. All candidates demonstrated good surface affinity and S73 revealed maximum hydrophobicity.

**TABLE 3:** Characterization of lactic acid bacteria candidates.

**Isolates**	**Sampling site**	**Morphology**	**Taxonomic identification**	**SCFA production (μmol/mL)**	**Auto-aggregation (%)**	**Co-aggregation (%)**	**Hydrophobicity (%)**
S1	Ileum	Rod	*L. salivarius*	96.13	42.31 ± 2.49	35.30 ± 2.17	65.57 ± 2.83
S26	Feces	Spherical	*P. acidilactici*	104.57	43.33 ± 2.05	33.33 ± 1.56	38.73 ± 1.58
S62	Crop	Rod	*L. agilis*	92.41	46.57 ± 0.91	37.12 ± 1.68	49.10 ± 1.75
S70	Feces	Rod	*L. salivarius*	96.51	41.35 ± 2.34	36.45 ± 3.30	45.97 ± 3.70
S73	Crop	Rod	*L. agilis*	124.18	53.98 ± 2.93	34.79 ± 1.57	70.13 ± 2.27

### Evaluation of Stress Tolerance

Tolerance against gastric pH conditions and small intestinal bile acids was tested to study the survival of the isolates during their passage through stomach and small intestine. Growth of both candidates was suppressed at pH 2, but survival increased at pH 3–4 (Table 4). Strain S73 seemed to tolerate lower pH slightly better than S1.

**TABLE 4:** Viability of final candidates under acidic conditions or bile challenge (log CFU/mL).

		**Incubation time**	**S1**	**Survival percentage (cell) (%)**	**S73**	**Survival percentage (cell) (%)**
Acid tolerance		0 h	8.14 ± 0.06	100	8.10 ± 0.042	100
	pH = 2	2 h	7.50 ± 0.00	22.91	7.86 ± 0.14	57.54
		4 h	5.77 ± 0.23	0.43	6.00 ± 0.20	0.79
		6 h	4.88 ± 0.09	0.05	5.22 ± 0.20	0.13
	pH = 3	2 h	7.70 ± 0.18	36.31	7.93 ± 0.07	67.61
		4 h	6.27 ± 0.05	1.35	7.21 ± 0.20	12.88
		6 h	6.01 ± 0.00	0.74	6.40 ± 0.01	2.00
	pH = 4	2 h	7.96 ± 0.25	66.07	7.99 ± 0.22	77.62
		4 h	7.07 ± 0.14	8.51	7.67 ± 0.13	37.15
		6 h	6.36 ± 0.01	1.66	6.86 ± 0.00	5.75
Bile tolerance		0 h	7.63 ± 0.07	100	8.02 ± 0.09	100
	2.45 mM	2 h	7.46 ± 0.05	20.89	7.60 ± 0.10	31.62
		4 h	6.69 ± 0.07	3.55	6.91 ± 0.03	6.46
		6 h	6.49 ± 0.07	2.24	6.74 ± 0.07	4.37
	7.35 mM	2 h	7.06 ± 0.03	8.32	7.31 ± 0.10	16.22
		4 h	6.71 ± 0.04	3.72	6.53 ± 0.05	2.69
		6 h	6.31 ± 0.08	1.48	6.39 ± 0.15	1.95
	12.25 mM	2 h	6.83 ± 0.01	4.90	6.850 ± 0.03	5.62
		4 h	6.03 ± 0.11	0.78	6.30 ± 0.07	1.58
		6 h	5.61 ± 0.12	0.30	5.83 ± 0.15	0.54
	17.15 mM	2 h	6.54 ± 0.08	2.51	6.70 ± 0.13	3.98
		4 h	5.93 ± 0.16	0.62	6.08 ± 0.05	0.95
		6 h	5.26 ± 0.12	0.13	5.69 ± 0.07	0.39

Both strains survived bile acid supplemented media well in the range from 2.45 to 7.35 mM (0.1% to 0.3% w/v) bile concentration, while 17.15 mM (0.7% w/v) concentration of bile exhibited stronger inhibitory effects (see Table 4). However, S1 generally showed slightly reduced tolerance in bile supplemented MRS medium compared to S73.

Both candidates demonstrated good resistance against increasing osmolarity (Table 5). Growth could still be detected until 8% NaCl. S1 showed a slightly better osmolarity resistance compared to S73.

**TABLE 5:** Growth capacity of final candidates under different osmotic pressures (final OD_690__nm_).

	**0% NaCl**	**2% NaCl**	**4% NaCl**	**6% NaCl**	**8% NaCl**	**10% NaCl**
S1	1.12 ± 0.09	0.99 ± 0.03	0.85 ± 0.01	0.60 ± 0.01^∗^	0.52 ± 0.06^∗^	0.15 ± 0.02
S73	0.99 ± 0.11	0.82 ± 0.08	0.70 ± 0.07	0.34 ± 0.03	0.26 ± 0.05	0.13 ± 0.06

*^∗^ = Significantly different between strains (*p* ≤ 0.05. Mann–Whitney test).*

### Antibiotic Susceptibility

The results in MIC test of selected candidates were interpreted according to the “Guidance on the assessment of bacterial susceptibility to antimicrobials of human and veterinary importance” documented by ESFA (2012). No resistance was observed against ampicillin, clindamycin, streptomycin and tetracycline. The strain S26 and S62 demonstrated resistance against gentamycin and kanamycin. S26 also indicated the resistance to chloramphenicol and erythromycin. S70 showed the resistance to kanamycin. The maximum susceptibility was observed against ampicillin and clindamycin. As the breakpoint of cefotaxime was not included in the documentation of ESFA, the results only revealed none of candidates was resistant to the cefotaxime at working concentration (8 μg/mL) of *ex vivo* model (Table 6).

**TABLE 6:** Susceptibility test of selected candidate strains to antibiotics.

	**S1**	**S26**	**S62**	**S70**	**S73**	**QC strain**
Ampicillin	<0.25^S^	2^S^	<0.25^S^	<0.25^S^	0.25^S^	0.5^S^
Chloramphenicol	4^S^	8^R^	4^S^	4^S^	2^S^	2^S^
Clindamycin	0.5^S^	<0.25^S^	0.25^S^	1^S^	1^S^	<0.25^S^
Erythromycin	0.25^S^	2^R^	0.25^S^	1^S^	1^S^	1^S^
Gentamycin	16^S^	64^R^	64^R^	8^S^	16^S^	8^S^
Kanamycin	32^S^	128^R^	128^R^	128^R^	32^S^	16^S^
Streptomycin	32^S^	32^S^	16^S^	8^S^	16^S^	16^S^
Tetracycline	4^S^	2^S^	4^S^	1^S^	4^S^	2^S^
Cefotaxime	0.25	2	2	1	1	2

*S = susceptible; R = resistant; QC strain = *L. reuteri* (DSM20016).*

### Adhesion and Competitive Adhesion Assay

**FIGURE 4:**
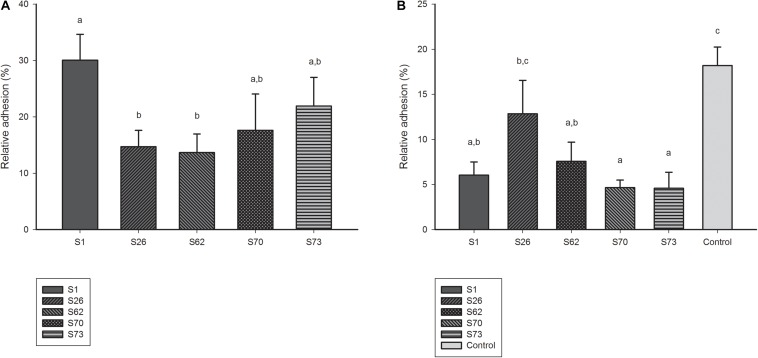
**(A)** Adhesion ability of lactobacilli candidates to Caco-2 cell monolayers. **(B)** Adhesion ability of model *E. coli* strain co-incubated with lactobacilli candidates to Caco-2 cell monolayers.

Among the five candidates tested in *ex vivo* model, S1 demonstrated the best adhesion capacity to human Caco-2 cell lines (Figure 4A). The competitive adhesion assay showed that the adhesion of *E. coli* model strain decreased significantly when co-incubated with all lactobacilli candidates except with strain S26 (Figure 4B).

## Discussion

Benefits of probiotics in animal nutrition are increasingly highlighted for their improvement of animal health by reducing the pathogenic bacterial load and the increase in animal performance (feed conversion, body weight gain) (Hong et al., 2005; Taras et al., 2005; Böhmer et al., 2006). Contrary to probiotics in human medicine/nutrition, probiotics in animal nutrition are often expected to specifically combat pathogenic bacteria (Markowiak and Śliżewska, 2018) which are of major concern in farm animal husbandry. Therefore, the selection of probiotics against those veterinary pathogens is always the preferred solution to improve animal health. In our current study, a combination of *in vitro* and *ex vivo* method is introduced to enable a rapid and comprehensive selection selection from massive probiotic LAB that are active against *E. coli*.

The scientific rationale for the focus on host specific lactobacilli as potential probiotics in this study is based on following reasons. Firstly, lactobacilli are known for their antagonistic activity against *E. coli* (Juven et al., 1991; Servin, 2004; Arena et al., 2018). Secondly, lactobacilli enjoy the generally-regarded-as-safe (GRAS) status as defined by the FAO or qualified presumption of safety (QPS) in the EU. Thirdly, several studies indicate that bacteria are expected with higher chance to colonize their hosts, if they are isolated from the same host (Yuki et al., 2000; Kwong et al., 2014). Thus, choosing lactobacilli as main target of isolation, the functional criterion (inhibition of *E. coli*) was combined with safety considerations (GRAS/QPS status) and high probability of viability in the intestinal tract. As highlighted by the FAO, a major potential safety concern of LAB can be their antibiotic resistance. In our antibiotic susceptibility assay on the five isolates used for *ex vivo* selection step, both final candidates (strain S1 and S73) demonstrated no resistance against recommended antibiotics, which increases the confidence of their use as safe feed additive in the future. Finally, host specificity has been deemed a favorable property for probiotic microorganisms (Saarela et al., 2000). Consequently, the robustness of potential probiotics against specific conditions of the GIT should be a pre-requisite for any candidate strain planned for *in vivo* colonization (Dicks and Botes, 2010; Fiocco et al., 2019). As the LAB candidates in this study were specifically designated to be used in broiler chicken, we followed this host-specific concept. To further ascertain the host specificity, an incubation temperature of 39°C has been applied to simulate the body temperature of broiler chicken and consequently yield mostly host-specific LAB isolates. In future applications of this method, this parameter can be changed depending on the host of interest.

The novelty in our procedure firstly lies in the number of screened isolates because enlarging the sample number simply enhances the chance to find probiotic isolates. Secondly, the validation of an *ex vivo* screening based on the survival of the model strain co-incubated with candidate LAB of interest under simulated *in vivo* conditions possibly predicts their functional activity in host animal. Thus, our experimental design combines three consecutive steps to progressively reduce the number of candidates with multiple criteria step by step (de Melo Pereira et al., 2018).

A literature search on isolation of probiotic bacteria revealed that the number of isolates ranges from 14 to 1150 isolates with the majority of publications using only 50 to 80 isolates (Robyn et al., 2012; Babot et al., 2014). To increase the number of potential isolates, a procedure allows both high throughput and easy method for detection of inhibitory activity is needed. Therefore, we designed a pre-screening step to identify potential candidates out of a massive sample pool (over 7000 isolates) by systematic processing instead of one-by-one treatment. This procedure also identified isolates which exhibit ease of growth and handling as a prerequisite for production of probiotics on a technical scale. Elimination of LAB that could not be cultivated under the relatively simple growth conditions of the assay probably led to a loss of many strains with potential inhibitory activity. However, fastidious growth conditions will inevitably lead to prohibitively high costs during later biomass production and thus, commercialization of the obtained isolates would be questionable.

During the selection progress, buffered or deacidified supernatants were used. It is known that lactic acid produced by lactobacilli can drastically reduce pH in media. Therefore, buffered/deacidified supernatants exclude pH-dependent inhibition via metabolites except for exceptionally high lactic acid production that overcomes the buffering capacity. On the other hand, a pH-independent inhibition involves different modes of action like production of bacteriocin or bacteriocin-like-substances (BLIS). In the present assay, both pH-dependent and pH-independent modes of action were considered.

The pre-screening step yielded 76 potential probiotic isolates out of the initial 4800 robust isolates (1.6%). These isolates were characterized in more depth via lag time extension of the *E. coli* model strain and lactic acid production. Since lag time extension is a key indicator in evaluating growth inhibition of microorganisms under adverse conditions (Swinnen et al., 2004; Rufián-Henares and Morales, 2008), it is often used to assess growth inhibition to various target microorganisms (Pereira et al., 2016; Alpaslan et al., 2017). The advantage of liquid based growth inhibition assays over agar diffusion assays is their sensitivity to observe subtle influences on growth (Fredua-Agyeman et al., 2017), and also their sample throughput. Therefore, we chose a microtiter plate-based assay to fit the purpose of rapid and efficient screening potentially probiotic LAB. Lactic acid production was considered as another inhibitory parameter, because exceptional lactic acid production *in vitro* may also yield high lactic acid production *in vivo*. For the studied isolates, it was also shown *in vitro* that other metabolites such as short chain fatty acids are negligible compared to lactic acid. The classic antagonism requires lactic acid to acidify the environment, which in turn inhibits growth of non-acid fast bacteria. However, lactic acid also exerts additional inhibition by disrupting the outer membrane to Gram-negative bacteria including *E. coli* (Alakomi et al., 2000). The use of lag time and lactic acid production indices enables the identification of isolates with the highest inhibitory activities. In the end, we chose five isolates among all candidates that exhibited the highest indices for both parameters.

In view of the complex environment in the intestinal tract, *in vitro* models cannot reflect antibacterial effects that may occur in the animal. It is always questionable whether probiotics inhabit or maintain their inhibitory activity *in vivo* well (Talpur et al., 2012). Consequently, *in vitro* tests are not able to mimic the complex intestinal matrix and truly reflect the inhibitory activity of probiotics in the GIT of animal (Saint-Cyr et al., 2016). However, *in vivo* experiments are costly and are subject to ethical considerations. *Ex vivo* assays try to find a compromise between both approaches. *Ex vivo* assays are advantageous due to higher replicate numbers and application of biological agents at defined concentrations. In the present study, although a two-fold dilution of digesta content may have led to a bias regarding the response of the biological matrix (partly hydrolyzed nutrients, metabolites, etc.). Nevertheless, part of the biological matrix was still intact and previous studies have shown that this *ex vivo* assay has the potential to mimic the bacterial response in intestinal contents. For instance, Starke et al. (2014) used a very similar *ex vivo* system on the response of intestinal bacteria to zinc and found that the system correctly predicted the bacterial response to zinc of later pig trials (Starke et al., 2014). Therefore, although the chosen *ex vivo* assay in this study had its limits, it still is a valuable tool to more closely elucidate possible inhibitory activity of bacterial isolates *in vitro*.

Compared to *in vitro* assays, the tested *Lactobacillus* candidates demonstrated different inhibitory effects against the *E. coli* model strain in the *ex vivo* model. Here, candidate S1 (*L. salivarius*) and S73 (*L. agilis*) showed the highest inhibitory potential. The other chosen LAB strains were not able to completely inhibit *E. coli* growth, although their *in vitro* performance was superior. Thus, the *ex vivo* assay has shown that it was indeed worthwhile to use an intermediate step before using probiotic isolates directly in feeding trials.

As recommended by WHO for selecting probiotics, host-related stress tolerance is usually considered as screening criteria in many studies (de Melo Pereira et al., 2018). The GIT induced stress was simulated *in vitro* according previous publications (Mongin et al., 1976; Lin et al., 2003; Lemme and Mitchell, 2008; Morgan et al., 2014; Nkukwana et al., 2015). Both *Lactobacillus* candidates demonstrated high viability in acidic incubations, maintained growth at pH 4, tolerated a wide range of bile concentrations and showed good resistance against high osmolality. Thus, a good survival in the GIT of the strains is expected and was predictable as they were isolated from the crop (S73) or ileum (S1) of broiler chicken. This also underscores the notion that host specific isolation increases the probability to isolate candidates with high survival rates in their respective host. However, four of the studied *Lactobacillus* isolates also inhibited *E. coli* adhesion in a commonly used intestinal model cell line, the Caco-2 cell lines, which may indicate a potential benefit of the selected candidates in competitive actions for intestinal niche. Metabolite production was also monitored and as expected, only minor amounts of acetate was found compared to production of lactic acid, while only traces of propionate and butyrate were present. This is in agreement with some previous studies (Imen et al., 2015). The level of propionate, butyrate and valerate was relatively low. This phenomenon might be because of being consumed as the energy for bacterial survival (Fernando et al., 2018).

The final two *Lactobacillus* spp. are currently used in feeding trials. Preliminary results indicate that the strains indeed modified the bacterial composition and activity metabolite concentration in the intestinal tract of broiler chicken (data not shown). Eventually, the employed combination of *in vitro* and *in vivo* combined method has the potential to isolate other probiotic bacteria with inhibitory activity against any other specific bacterium, as long as a specific biomarker for pathogens (for instance antibiotic resistance) is available. With modification regarding growth condition as well as the detection method for the bacterium in question, the described method can be expanded to other probiotic species for a targeted search against specific microbes. This gives the method a general applicability in a more comprehensive and rapid way.

## Conclusion

In the present study, a novel three-strep rapid screening method consisted is reported for the isolation of probiotic LAB against a target *E. coli*. It includes a pre-screening step as an effective filter of a massive isolate pool and easy-handling of the isolates for later technical scale cultivation; an *in vitro* selection step to assure the correct choice of the most active isolates and finally, an *ex vivo* assay to confirm probiotic function of the candidates *in vivo*. As a proof-of-principle we have chosen lactobacilli as antagonist to *E. coli*, but the system can be employed to screen any cultivable probiotic bacterium and its inhibitory activity against any cultivable bacterium with a specific biomarker.

## Data Availability Statement

The raw data supporting the conclusions of this manuscript will be made available by the authors, without undue reservation, to any qualified researcher.

## Ethics Statement

Ethical approval Samples were taken from studies that were conducted in accordance with the German Animal Welfare Act (TierSchG) and approved by the local state office of occupational health and technical safety “Landesamt für Gesundheit und Soziales, Berlin” (LaGeSo Reg. Nr. T 0162/16 and A 0100/13).

## Author Contributions

HR, WV, and JZ organized the whole study. HR and WV developed the protocol of the screening method. HR performed the experiments, analyzed data and wrote the first version of the manuscript. E-MS contributed to the selection of target *E.coli* and provided the basic information about the bacteria. FG contributed to the animal trial and sampling. WV, JZ, E-MS, and FG revised the manuscript. All authors read and approved the final manuscript as submitted and agreed to be accountable for all aspects of the work.

## Conflict of Interest

The authors declare that the research was conducted in the absence of any commercial or financial relationships that could be construed as a potential conflict of interest.
