# Corrigendum: Screening of Host Specific Lactic Acid Bacteria Active Against *Escherichia coli* From Massive Sample Pools With a Combination of *in vitro* and *ex vivo* Methods

**DOI:** 10.3389/fmicb.2020.00459

**Published:** 2020-03-20

**Authors:** Hao Ren, Eva-Maria Saliu, Jürgen Zentek, Farshad Goodarzi Boroojeni, Wilfried Vahjen

**Affiliations:** Institute of Animal Nutrition, Freie Universität Berlin, Berlin, Germany

**Keywords:** probiotics, lactic acid bacteria, host-derived, effective screening, *E. coli*, *ex vivo* model, massive sample pool

In the original article, there was a mistake in Figure 3 as published. The authors reversed the order of Figures 3A,B by mistake when uploading the figures. The corrected figure appears below.

**Figure 3:**
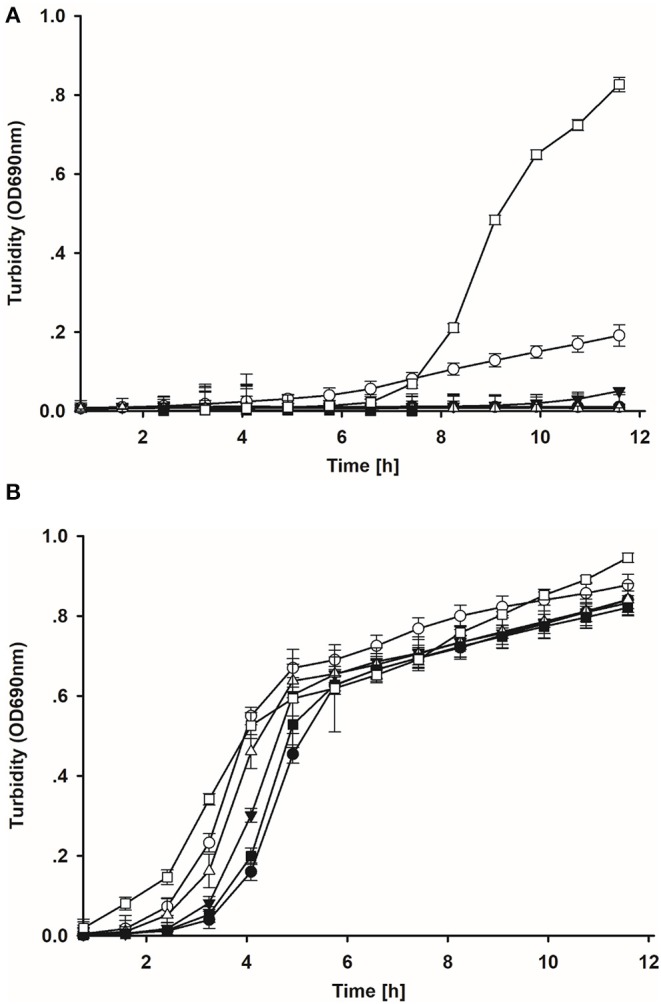
**(A)** Growth of the model *E. coli* strain after *ex vivo* co-incubation with candidate lactic acid bacteria isolates for 8 h in crop contents. Filled circle = S1; open circle = S26; filled down triangle = S62; open downward triangle open diamond = S70; filled square = S73; open square = control. **(B)** Growth of the model *E. coli* strain after *ex vivo* co-incubation with candidate lactic acid bacteria isolates for 8 h in jejunum contents. Filled circle = S1; open circle = S26; filled down triangle = S62; open downward triangle open diamond = S70; filled square = S73; open square = control.

The authors apologize for this error and state that this does not change the scientific conclusions of the article in any way. The original article has been updated.

