# Supplementary material for: Screening of Host Specific Lactic Acid Bacteria Active Against Escherichia coli From Massive Sample Pools With a Combination of in vitro and ex vivo Methods
Source: Front Microbiol. 2019 Nov 21;10:2705. doi: 10.3389/fmicb.2019.02705 (PMC6882376; doi:10.3389/fmicb.2019.02705)
Supplement: Supplementary file 1 [file Table_1.docx]

Supplementary Material

## Supplementary Figures


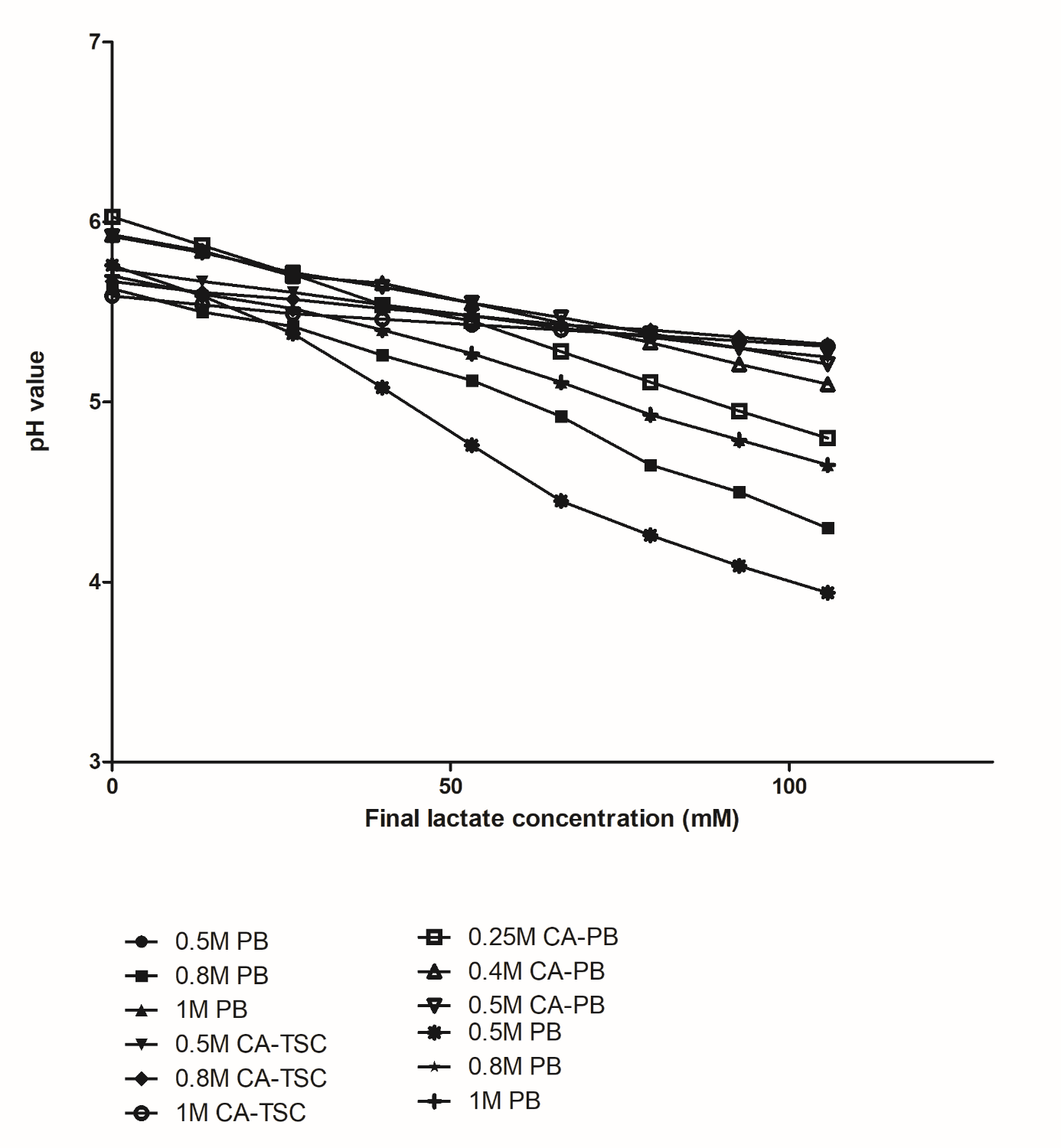


**Supplementary Figure 1. buffering capacity of different buffer system**

Note: PB: phosphate buffer; CA-PB: citric acid- phosphate buffer; CA-TSC: citric acid-tri sodium citrate buffer.


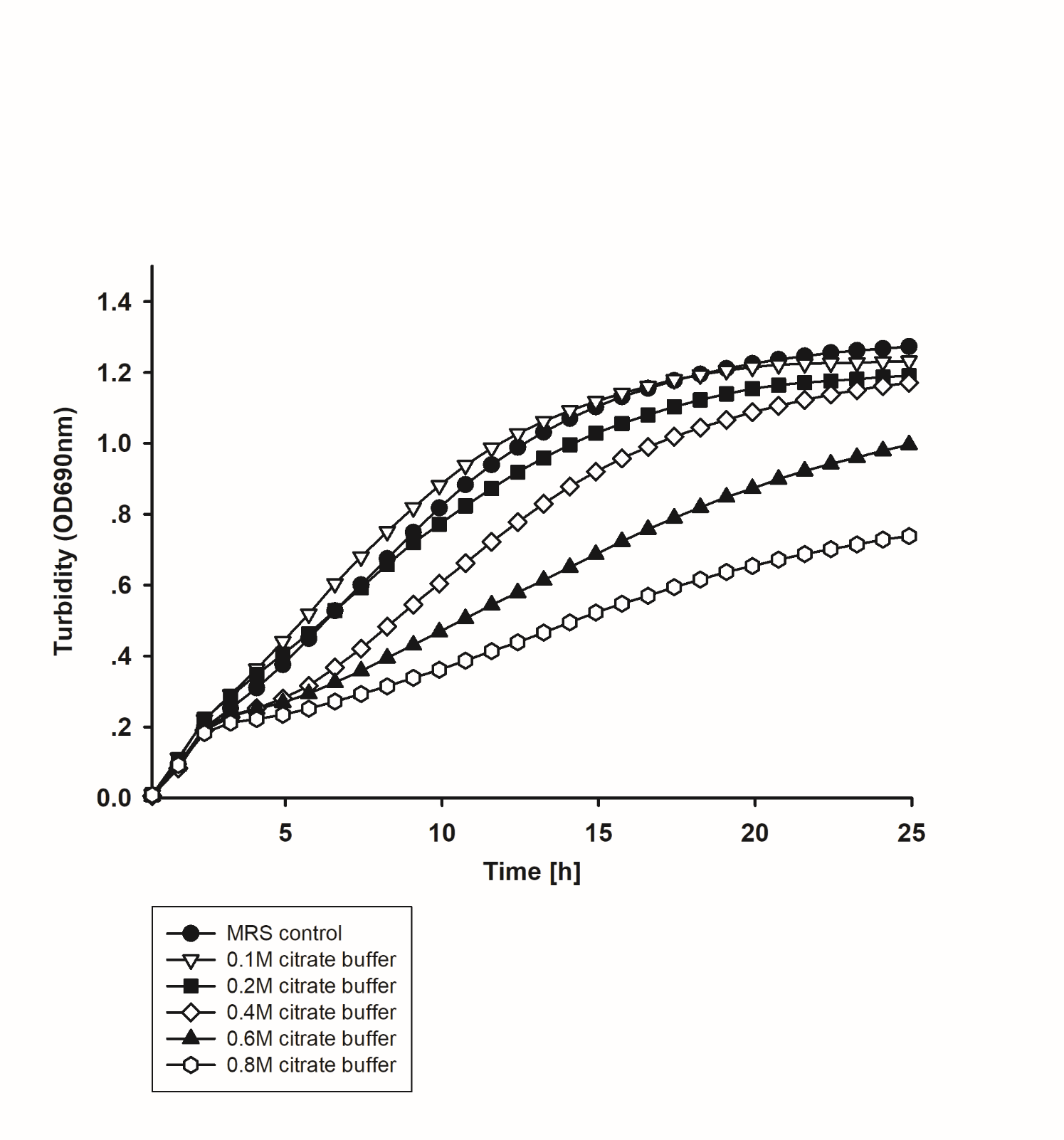


**Supplementary Figure 2. Growth profile of bacteria under buffering stress**

The *E. coli* model strain was inoculated to medium supplemented with CA-TSC buffer at increasing concentrations to find ideal survival of the indicator strain.


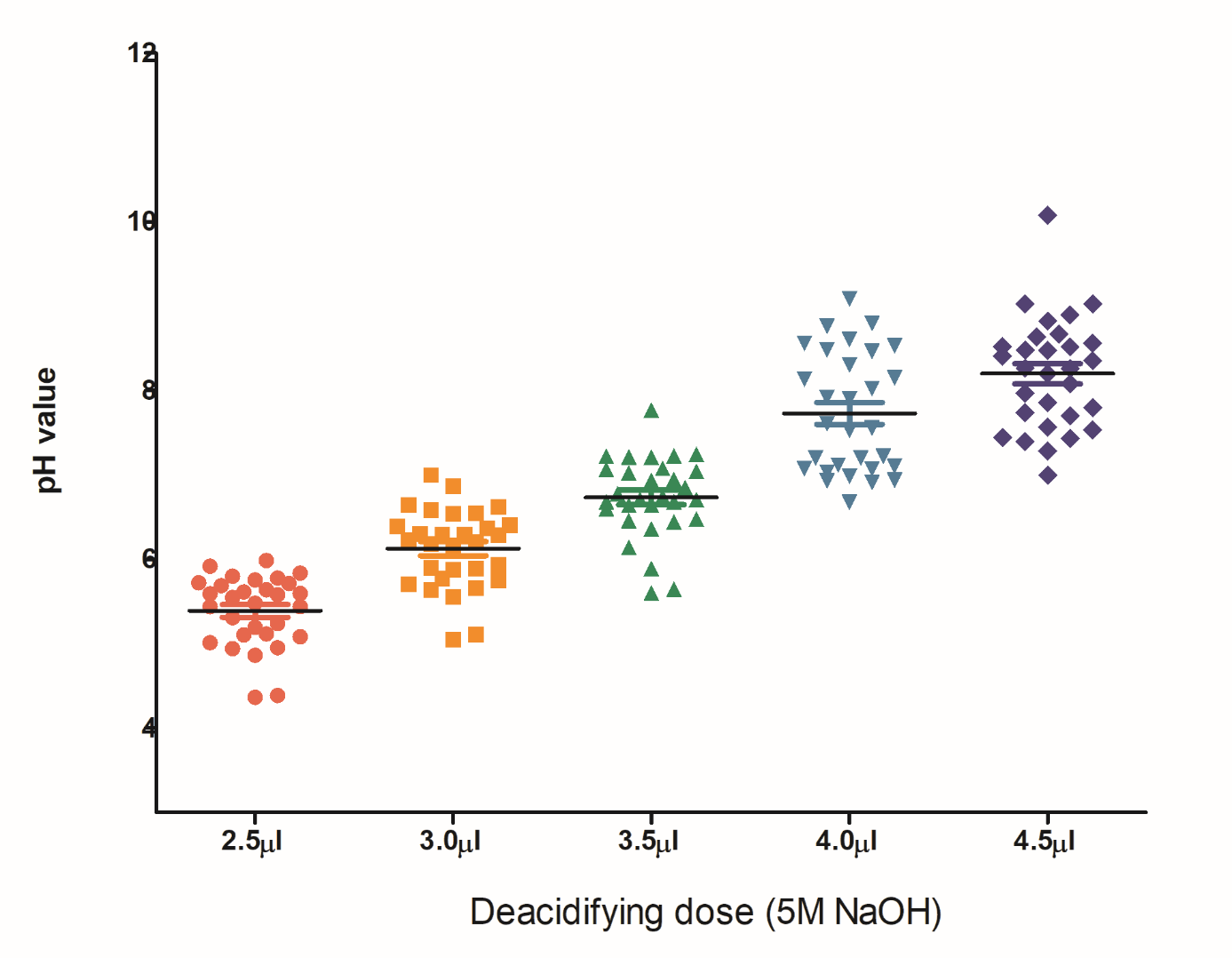


**Supplementary Figure 3. Optimization of deacidifying dose of NaOH**

The supernatants of 30 random isolates were treated with 5M NaOH at increasing volumes. The pH was measured after mixing to indentify the optimal deacidifying volume.


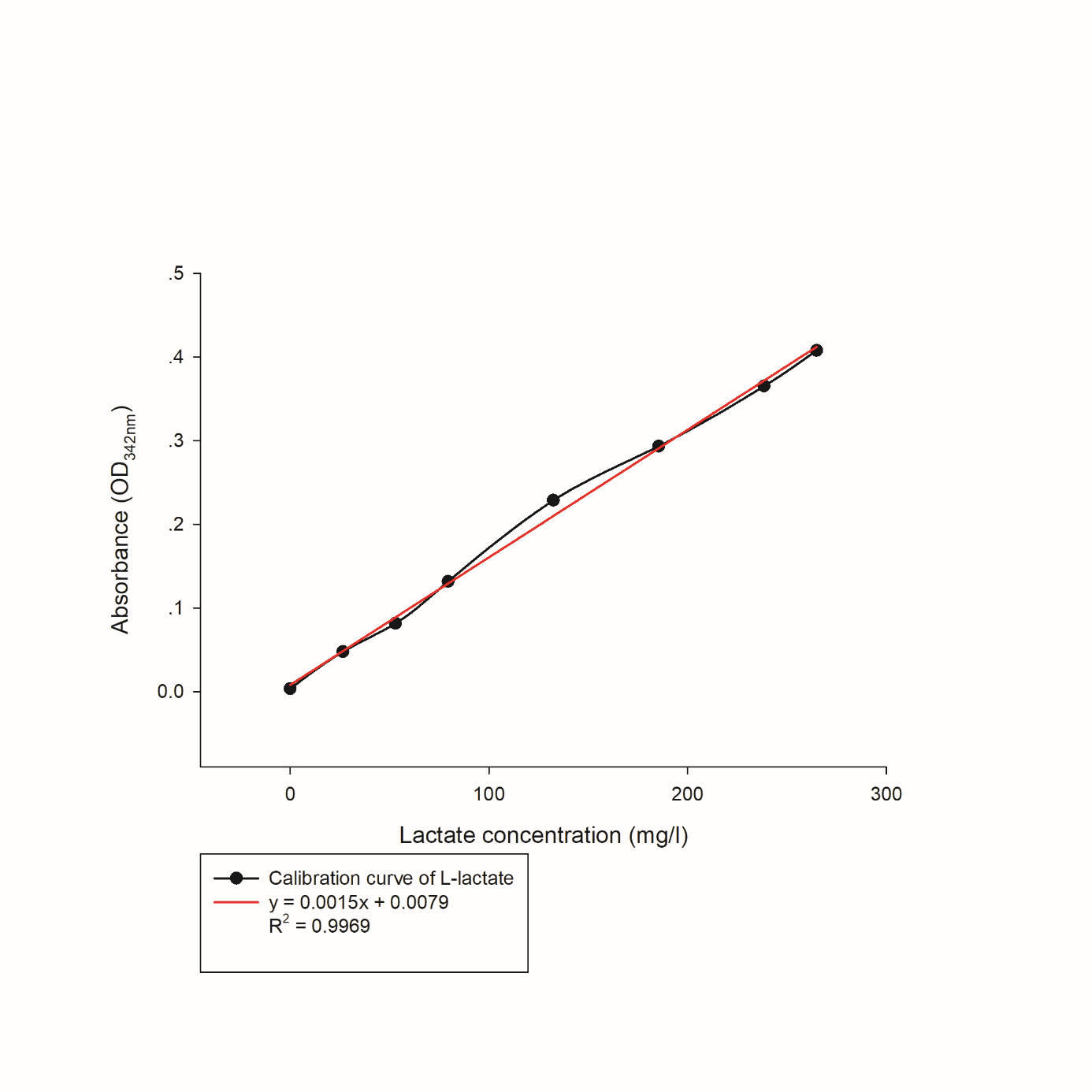


**Supplementary Figure 4. Calibration curve of L-lactate for the determination of L-Lactate production in spent medium**

**
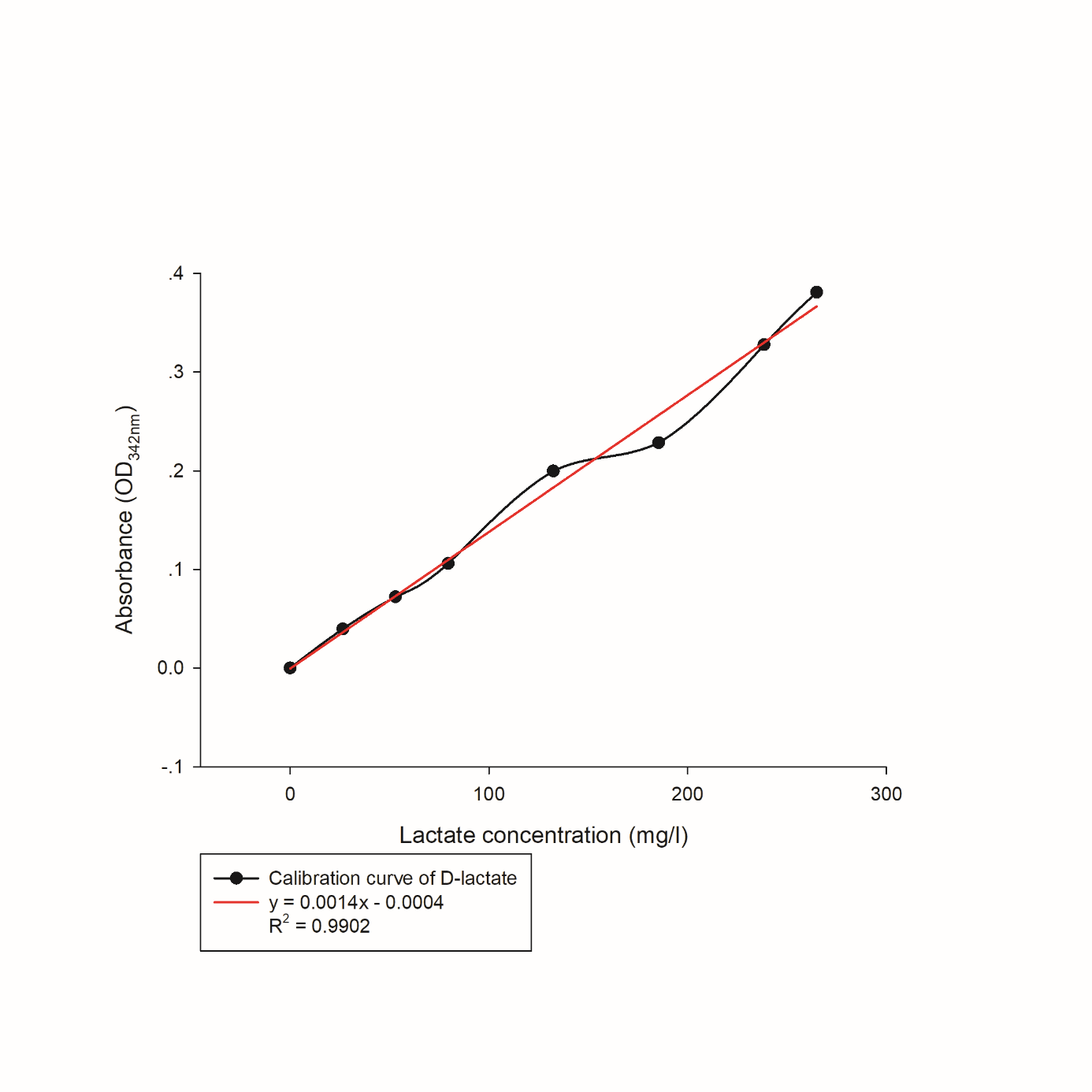
**

**Supplementary Figure 5. Calibration curve of D-lactate for the determination of D-Lactate production in spent medium**

The calibration curve of pure L/D-lactate was established to measure the lactate yield in the supernatants of candidates.

Supplementary Tables

Supplemental Table 1: Average lag time of the model *E. coli* strain during incubation with isolate supernatants (no. 1-76) in the second step of the assay

| Running Nr | Mean ± SD (h) |
| --- | --- |
| 1 | 8.572 ± 1.160 |
| 2 | 7.809 ± 0.924 |
| 3 | 7.691 ± 0.965 |
| 4 | 7.720 ± 0.944 |
| 5 | 7.931 ± 1.005 |
| 6 | 8.028 ± 1.046 |
| 7 | 8.090 ± 0.993 |
| 8 | 8.209 ± 0.951 |
| 9 | 7.878 ± 0.929 |
| 10 | 7.607 ± 1.037 |
| 11 | 7.513 ± 0.970 |
| 12 | 7.600 ± 0.952 |
| 13 | 7.834 ± 0.978 |
| 14 | 7.816 ± 1.053 |
| 15 | 7.922 ± 1.013 |
| 16 | 8.199 ± 1.028 |
| 17 | 7.853 ± 0.944 |
| 18 | 7.723 ± 1.056 |
| 19 | 7.676 ± 1.001 |
| 20 | 8.294 ± 1.300 |
| 21 | 8.237 ± 1.579 |
| 22 | 8.237 ± 1.659 |
| 23 | 7.657 ± 0.960 |
| 24 | 7.836 ± 1.005 |
| 25 | 8.157 ± 0.854 |
| 26 | 8.010 ± 0.865 |
| 27 | 7.677 ± 0.943 |
| 28 | 8.056 ± 1.373 |
| 29 | 7.922 ± 1.138 |
| 30 | 7.469 ± 0.974 |
| 31 | 8.148 ± 1.321 |
| 32 | 8.539 ± 1.365 |
| 33 | 7.908 ± 1.226 |
| 34 | 7.552 ± 1.267 |
| 35 | 7.515 ± 1.259 |
| 36 | 7.783 ± 1.271 |
| 37 | 7.772 ± 1.372 |
| 38 | 7.755 ± 1.374 |
| 39 | 7.792 ± 1.251 |
| 40 | 8.099 ± 1.372 |
| 41 | 7.953 ± 1.357 |
| 42 | 8.052 ± 1.202 |
| 43 | 7.977 ± 1.152 |
| 44 | 7.663 ± 0.845 |
| 45 | 8.111 ± 1.211 |
| 46 | 8.531 ± 1.463 |
| 47 | 8.318 ± 1.192 |
| 48 | 8.126 ± 1.298 |
| 49 | 8.344 ± 1.051 |
| 50 | 7.916 ± 0.764 |
| 51 | 7.749 ± 0.953 |
| 52 | 7.729 ± 0.901 |
| 53 | 7.822 ± 0.824 |
| 54 | 8.037 ± 0.929 |
| 55 | 7.751 ± 0.965 |
| 56 | 8.346 ± 1.138 |
| 57 | 7.627 ± 0.787 |
| 58 | 7.880 ± 0.817 |
| 59 | 7.797 ± 0.886 |
| 60 | 7.802 ± 0.804 |
| 61 | 7.681 ± 0.804 |
| 62 | 8.087 ± 0.795 |
| 63 | 8.016 ± 1.152 |
| 64 | 7.961 ± 0.747 |
| 65 | 7.629 ± 0.860 |
| 66 | 7.879 ± 0.796 |
| 67 | 7.753 ± 0.923 |
| 68 | 7.679 ± 0.827 |
| 69 | 8.258 ± 1.034 |
| 70 | 8.687 ± 0.829 |
| 71 | 8.135 ± 1.023 |
| 72 | 8.441 ± 0.981 |
| 73 | 8.865 ± 1.390 |
| 74 | 8.426 ± 1.200 |
| 75 | 8.116 ± 0.918 |
| 76 | 8.462 ± 1.263 |
| Control | 6.291 ± 0.874 |
